# Some Experiences of Training-School Life

**Published:** 1918-07-20

**Authors:** 


### THE MATRONS' AND SISTERS' DEPARTMENT.

### SOME EXPERIENCES OF TRAINING-SCHOOL LIFE.

BY A CERTIFICATED TRAINED NURSE.

### I. Does Training Harden Character?

One of the remarks that earliest impressed themselves upon the mind of the writer within the first week or two of hospital life came from an earnest and enthusiastic probationer of about a month's standing, and was to the effect that she was "dreadfully disappointed in the women" (*i.e.*, the sisters and nurses). Another remark which has, to the writer's great distress, been frequently made during the past year, recalls this early unbiased, if inexperienced, judgment, and gives one "furiously to think." It is that the soldier in hospital has a far higher opinion of the nursing V.A.D. than of the trained nurse or sister. He is afraid of the latter and inclined to dread her appearing; he welcomes the coming of the former. Why? Because the untrained and inexperienced junior still possesses the instinctive sympathy, the loss of which has robbed the fully-trained nurse of the human womanliness which every soldier has a right to expect from those who tend him. Soldiers have a crude way of expressing themselves sometimes, and the actual words used were such as to provoke an instant and indignant denial and the assertion that this was an isolated instance. Unfortunately, however, the same opinion was repeated from so many widely different quarters that it was obviously not without foundation. Again, an old woman in hospital was once thanking a new probationer for some gentleness of touch and unbidden service, and said, "Ah, nurse, you're very gentle and thoughtful now, but in two years' time you'll be as hard as the rest of them."

The contradiction of a truth cannot make it less true, and it therefore behoves us to search for the cause and the remedy. One of the most interesting features of the annual meeting of the College of Nursing was the discussion in Conference of the educational side of the nurse's career, and the recognition by the speakers that the present system is not ideal. Once this fact is recognised, the way lies open to clearer thinking.

When a hospital board receives a probationer, what does it propose to do with her? The most obvious reply is probably "Make her into a good nurse." Now therein lies a certain amount of ambiguity. How often, both in hospital and outside of it, does one hear phrases like these:—"A good nurse and such a nice woman"; or "She's awfully hard, but she's a good nurse"; or even "She's an excellent sister, but the patients can't bear her"! These assertions should be in the one case tautological and in the other contradictions in terms, for nursing is almost the only profession in which the individual cannot be separated from her work. A lawyer may be two entirely different beings in his professional and in his private life: so also may a soldier, an architect or engineer, an artist or musician; so also may even a surgeon or physician. But a nurse's work is so essentially herself that you cannot really have a "good nurse" unless she expresses certain qualities over and above those which nowadays tend to be regarded as purely professional. It is undoubtedly true that the nursing profession produces some of the best women that have ever lived, but it is probably also true that the good in them had already been so fully developed as to be able to withstand the hardening influences that hitherto have been indubitably present in the average hospital training. Let us endeavour to discern exactly what these hardening influences are, and whether they are an inevitable accompaniment of training; if not, how they may be removed; and if so, in what direction we may look for an influence so strongly counteracting as to nullify their evil effects.

(*To be continued.*)

